# Rift Valley Fever, Sudan, 2007 and 2010

**DOI:** 10.3201/eid1902.120834

**Published:** 2013-02

**Authors:** Imadeldin E. Aradaib, Bobbie R. Erickson, Rehab M. Elageb, Marina L. Khristova, Serena A. Carroll, Isam M. Elkhidir, Mubarak E. Karsany, AbdelRahim E. Karrar, Mustafa I. Elbashir, Stuart T. Nichol

**Affiliations:** Author affiliations: University of Khartoum, Khartoum, Republic of the Sudan (I.E. Aradaib, I.M. Elkhidir, A.E. Karrar, M.I. Elbashir);; Centers for Disease Control and Prevention, Atlanta, Georgia, USA (B.R. Erickson, M.L. Khristova, S.A. Carroll, S.T. Nichol);; Federal Ministry of Health, Khartoum (R.M. Elageb, M.E. Karsany)

**Keywords:** Molecular characterization, disease outbreaks, Rift Valley fever, genomic analysis, phylogenetic relationship, Sudan, viruses, vector-borne infections

## Abstract

To elucidate whether Rift Valley fever virus (RVFV) diversity in Sudan resulted from multiple introductions or from acquired changes over time from 1 introduction event, we generated complete genome sequences from RVFV strains detected during the 2007 and 2010 outbreaks. Phylogenetic analyses of small, medium, and large RNA segment sequences indicated several genetic RVFV variants were circulating in Sudan, which all grouped into Kenya-1 or Kenya-2 sublineages from the 2006–2008 eastern Africa epizootic. Bayesian analysis of sequence differences estimated that diversity among the 2007 and 2010 Sudan RVFV variants shared a most recent common ancestor circa 1996. The data suggest multiple introductions of RVFV into Sudan as part of sweeping epizootics from eastern Africa. The sequences indicate recent movement of RVFV and support the need for surveillance to recognize when and where RVFV circulates between epidemics, which can make data from prediction tools easier to interpret and preventive measures easier to direct toward high-risk areas.

## Abstract 2

Viral sequences analyzed indicate recent virus movement and support the need for surveillance.

Rift Valley fever (RVF) is a mosquito-borne viral disease that typically occurs in various areas of sub-Saharan Africa, where virus activity varies from a low-level enzootic cycle to explosive outbreaks covering large areas. Periodically, Rift Valley fever virus (RVFV) spreads to other areas, including northward into Egypt in 1977 and eastward across the Red Sea into Saudi Arabia and Yemen in 2000 (*1*–*7*). How RVFV travels is unclear but probably involves movement of infected livestock or mosquitoes.

Flooding and filling of shallow depressions (*damboes*) during unusual weather events create ideal conditions for emergence of RVFV-infected mosquitoes. The primary vector is *Aedes* spp. floodwater mosquitoes, which transmit the virus transovarially, so infected mosquito eggs lay dormant, ready to hatch when surface water levels rise. The infected mosquitoes feed on livestock, causing high viremia, and provide a way for RVFV to 1) infect secondary vector mosquito species (e.g., *Culex* spp. mosquitoes), which can further transmit the virus to other animals and humans, and 2) infect humans who contact infected animal tissues and blood (*8*,*9*).

RVF in livestock can devastate agricultural communities; it causes almost 100% mortality rates among young animals and high abortion rates among livestock. Most RVF in humans is asymptomatic or a mild febrile illness; only 1%–2% of cases progress to more severe disease, such as acute hepatitis, encephalitis, retinitis, and/or a hemorrhagic syndrome; case-fatality rates among hospitalized patients reach 10%–20% (*10*–*12*). When outbreaks cover a wide geographic area, hundreds of thousands of livestock are affected, leading to tens of thousands of human infections and hundreds of hospitalizations.

The first reported outbreak of RVF in Sudan occurred in 1973 in sheep and cattle in White Nile State; shortly thereafter, RVFV was isolated from a herd of cattle in northern Khartoum (*2*,*3*,*13*). Serologic surveys have detected RVFV antibodies in domestic livestock (*14*,*15*) and in humans from different Sudanese states, including Nile, Khartoum, Kassala, El Gezira, Sennar, and White Nile (*16*–*18*). A recent seroepidemiologic survey reported a high prevalence of RVFV IgG among febrile patients admitted to New Halfa Hospital in Kassala State (*19*). New Halfa is an extensively irrigated agricultural area ≈500 km east of Khartoum. Although the presence of IgG does not indicate recent infection, it does suggest considerable circulation of RVFV in the area at some time in the past.

In late fall and early winter 2007, a large outbreak of RVF, characterized by abortion storms among domestic livestock and febrile hemorrhagic illness in humans, was reported in several Sudanese states (*20*). Estimates suggested ≈75,000 human infections, similar to the number estimated to have occurred in Kenya around this time (*21*). The clinical descriptions of severe RVF cases from the 2007 outbreak are similar to those reported from earlier outbreaks (*22*–*24*). Several human RVF cases also were reported in 2010 from the El Gezira State after an increase in abortions among ewes and does; however, the outbreak appears to have been limited geographically, and little information is available about the outbreak (I.E. Aradaib, pers. comm.).

The large, well-documented RVF outbreak in Kenya and Tanzania in 2006–2007, with subsequent spread to Madagascar by 2008 (*1*,*8*,*25*–*30*), was characterized as a steady expansion of many circulating strains of RVFV from 2 sublineages (Kenya-1 and Kenya-2) rather than the introduction and spread of a single strain (*5*,*25*,*26*,*31*). However, to our knowledge, no information is available about the genetic lineages of RVFV strains circulating in Sudan during this time and in subsequent years. The purpose of this study, therefore, was to generate whole-genome sequences of RVFV associated with the 2007 and 2010 RVFV outbreaks in Sudan. Comparison of these sequences with those derived from known strains from eastern Africa and Egypt may provide insight into the origins of Sudan RVFV strains and whether recent outbreaks in Sudan resulted from single or multiple virus introductions.

## Materials and Methods

### Case Definitions

A suspected RVF case was defined as fever with or without hemorrhagic or neurologic signs, jaundice, or retinitis, in a person who had a history of contact with infected animals (meat, body fluid, aborted fetus). For the 2007 outbreak, the case definition was restricted to illness in persons seeking care during October–December 2007; for the 2010 outbreak, the definition was restricted to persons from the El Gezira State. A confirmed RVF case was defined as laboratory-confirmed acute or recent RVFV infection by positive RVFV IgM detection and/or positive reverse transcription PCR (RT-PCR).

### Specimen Collection and Preparation

**Figure 1:**
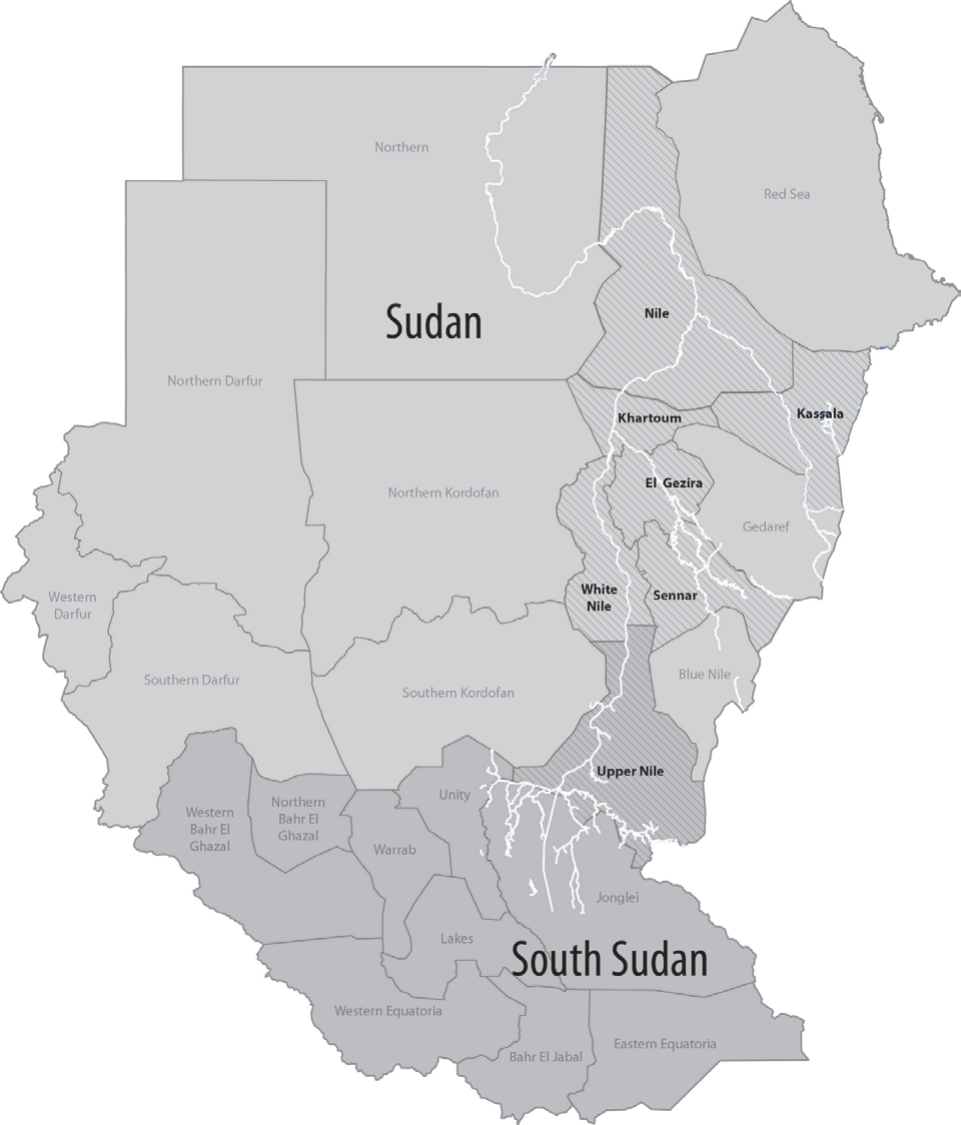
Sudan and South Sudan. States with confirmed Rift Valley fever cases are in **boldface**. Light gray indicates Sudan; dark gray indicates South Sudan. The Nile, White Nile, and Blue Nile Rivers are depicted in white, and other bodies of water were removed for clarity.

Serum was collected from a total of 256 suspected RVF case-patients. During October–December 2007, a total of 156 samples were collected from different Sudanese states: Nile, Khartoum, Kassala, El Gezira, White Nile, Sennar, and Upper Nile (now in the Republic of South Sudan; Figure 1). The remaining 100 samples were collected in October 2010 from the El Gezira State in central Sudan (Figure 1). After informed consent was obtained, a blood specimen was collected, and each participant was interviewed. A standardized questionnaire was used to collect information about demographic characteristics, such as age, sex, state of residence, and sample collection date for the 2007 suspected case-patients.

Blood samples were allowed to clot, and serum was separated and sent to the National Medical Health Laboratory (Khartoum, Sudan) for serologic diagnostic screening. RNA was extracted from serum by using a QiaAmp viral RNA Mini Kit (QIAGEN, Hilden, Germany) and sent to the Molecular Biology Laboratory, University of Khartoum, for conventional RT-PCR amplification. Aliquots of selected serum samples in Trizol reagent buffer at a 1:5 ratio were shipped to the Centers for Disease Control and Prevention (CDC; Atlanta, GA, USA) for whole-genome sequence analysis and subsequent phylogenetic studies.

### Serologic Diagnostic Tests

Virus isolation was not attempted because of a lack of a biosafety level 3 facility at the University of Khartoum and Ministry of Health. Therefore, virus identification depended solely on serologic testing and conventional RT-PCR amplification results. In Sudan, serum was screened for RVFV IgM by using the ELISA kit from Biologic Diagnostic Supplies Ltd (Johannesburg, South Africa) following the manufacturer’s instructions.

### RVFV Molecular Diagnostic Tests

Selection of RVFV primers was based on a highly conserved fragment of the small (S) RNA segment of the Smithburn RVFV strain (GenBank accession no. GQ862371) and multiple published RVFV sequences and by using BioEdit software (www.mbio.ncsu.edu/bioedit/bioedit.html). A forward primer RVF1 (5′-AAG CCA TAT CCT GGC CTC TT-3′) and a reverse primer RVF2 (5′-TCC AGT TGT TTC TCC CCA TC-3′) were used to amplify a 390-bp primary PCR product.

RVFV RNA was amplified with conventional RT-PCR by using a SuperScript III One-Step RT-PCR System with Platinum Taq High Fidelity (Invitrogen, Carlsbad, CA, USA), as described (*32*). Crimean-Congo hemorrhagic fever virus and dengue virus RNA were used as negative-control templates. Thermal profiles were performed on a Techne PHC-2 thermal cycler (Techne, Princeton, NJ, USA); reactions were incubated for 30 min at 50°C, followed by 40 cycles of 95°C for 1 min, 56°C for 30 sec, and 72°C for 45 sec, and a final incubation at 72°C for 10 min.

After amplification, the 390-bp PCR products were purified by using the QIAquick PCR Purification Kit (QIAGEN, Hilden, Germany) and shipped to a commercial company (Seqlab, Göttingen, Germany) for partial sequencing. Sequences were edited by using BioEdit software, and BLAST (http://blast.ncbi.nlm.nih.gov) was used to confirm the identity of the generated sequences.

In addition to the RT-PCR described above, the conventional RT-PCR described by Aradaib et al. (*33*) and TaqMan-based real-time RT-PCR described by Drosten et al. (*34*) also were used. Samples were negative for dengue virus, Crimean-Congo hemorrhagic fever virus, and flaviviruses.

### RVFV Whole-Genome Sequencing

Twelve serum samples from the 2007 outbreak and 18 serum samples from the 2010 RVF outbreak, all positive for RVFV by conventional RT-PCR, were sent to CDC for complete S, medium (M), and large (L) segment amplification and sequencing as described (*25*,*26*,*31*). Serum samples were sent in Trizol reagent; after a chloroform extraction, RNA was extracted by using the RNeasy Mini Kit (QIAGEN, Valencia, CA, USA). The SuperScript III One-Step RT-PCR System with Platinum Taq High Fidelity (Invitrogen) was used in accordance with the manufacturer’s instructions by using segment-specific primers (*31*). The samples were cycled as follows: 51°C (S, M) or 56°C (L) for 30 min; 94°C for 2 min; 40 cycles at 94°C for 15 sec, 56°C (S) or 46°C (M, L) for 30 sec, 68°C for 2 min (S) or 5 min (M, L); and a final extension at 68°C for 5 min.

RT-PCR products were purified with ExoSAP-IT (USB Corporation, Cleveland, OH, USA) before sequencing by using BigDye Terminator version 3.1 (Applied Biosystems, Carlsbad, CA, USA) and the ABI 3730XL automated DNA sequencer (Applied Biosystems). For sequence gaps, specific internal primers were used to amplify smaller products for additional sequencing. Thirteen complete S segments (GenBank accession nos. JQ820472–82, JQ840745–6), 5 complete M segments (GenBank accession nos. JQ820487–91), and 4 complete L segments (GenBank accession nos. JQ820483–6) were generated for phylogenetic analysis.

### Phylogenetic Analysis

SeaView software (http://pbil.univ-lyon1.fr/software/seaview3.html) was used to align each complete S, M, and L RVFV segment sequence obtained from this study with those available in GenBank as of December 2011. Bayesian coalescent analyses were performed by using the BEAST and Tracer software packages (*35*). The relaxed uncorrelated exponential clock (*36*) and Bayesian Skyline population size models were chosen for the S, M, and L segments on the basis of recent analyses conducted by Carroll et al. (*25*). Runs consisted of 100 million to 240 million generations to ensure effective sample sizes of at least 200. Maximum clade credibility trees were summarized with TreeAnnotator and were depicted by using FigTree (*35*).

### Statistical Analysis

Data were analyzed by using Statistical Package for Social Sciences (SPSS; IBM, Armonk, NY, USA) version 17 for Windows (Microsoft, Redmond, WA, USA). Chi-square tests were used to compare ELISA and RT-PCR data, association between RVFV infection and time period, and sex. According to data distribution, mean age was compared with RVFV infection by using the Mann-Whitney U test.

## Results

### Suspected RVF Case-patients, 2007

**Figure 2:**
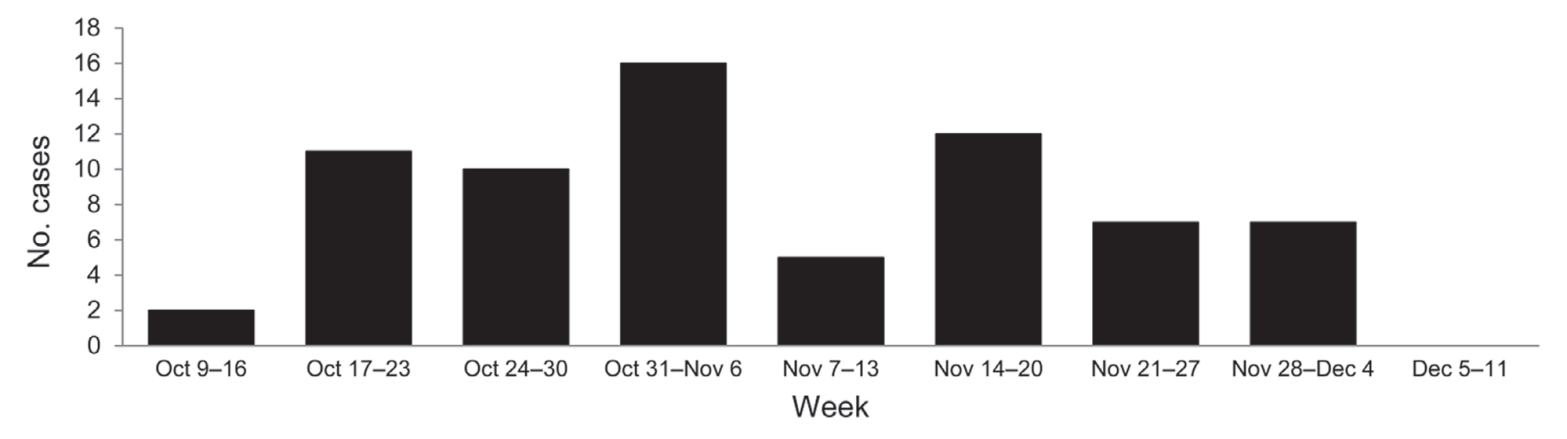
Laboratory-confirmed Rift Valley fever cases, Sudan, 2007.

The outbreak occurred during October 9–December 4, 2007; most (16) cases were reported during the fourth week of the outbreak (Figure 2). Serum samples from 156 persons whose illness fit the clinical case definition were tested for RVFV infection by IgM ELISA, which detects an early antibody response to infection, and/or RT-PCR, which detects viral RNA during acute infection. Seventy-eight (50%) of these suspected case-patients were positive for RVFV infection by either test; 23 were positive by IgM ELISA only, 52 by RT-PCR only, and 3 by both tests. Ninety-four (60%) case-patients were male, of whom 50 had positive RVFV results; similarly, 28 of the 62 female suspected case-patients had positive RVFV results. When using the χ^2^ test, we found no statistically significant differences between sex and RVF infection. The age distribution of suspected case-patients was 1.5–85 years (mean 28 years; 19.7 years ± SD). Most (46) suspected case-patients were from Khartoum State, 37 from El Gezira, 34 from Kassala, 24 from White Nile, 6 from Sennar, 6 from Upper Nile, and 3 from Nile State (Figure 1).

### Suspected RVF Case-patients, 2010

The 2010 outbreak was restricted to a rural area in El Gezira State in central Sudan (Figure 1). One hundred serum samples were collected from suspected case-patients. Suspected RVFV samples were tested solely by RT-PCR, and 18 samples were positive.

### Whole-Genome Sequencing

Serum from patients with positive test results for RVFV by conventional RT-PCR was subjected to whole-genome sequencing. Twelve samples from 2007 and 18 samples from 2010 were used to attempt generation of complete S, M, and L segment sequences (Table). From the 2007 samples, 6 complete S segments, 1 complete M segment, and 1 complete L segment were amplified and sequenced; 7 S, 4 M, and 3 L segments were obtained from the 2010 samples. Of these sequences, S segments from samples 2V, 1, and SP from 2007 were identical. S segments from samples 7 and 85 from 2010 also were identical, and the M segment of these samples contained a single base change. Sample 4 from 2010 and sample 77 from 2007 also shared an identical S segment sequence. The generated sequences represent Khartoum, El Gezira, and White Nile States (Table, Figure 1).

**Table:** Full-genome Rift Valley fever virus sequences and GenBank accession numbers for Sudan Rift Valley fever virus strains, Sudan

Year collected, strain	State	Date	GenBank accession no.		
			Full-length S	Full-length M†	Full-length L†
2007					
1	Khartoum	Oct	JQ840745		
2V	White Nile	2007*	JQ820472	JQ820490	JQ820483
77	Khartoum	Nov	JQ820482		
133	Khartoum	Nov	JQ820478		
SP	White Nile	2007*	JQ820479		
B	Unknown	2007*	JQ840746		
2010					
4	El Gezira	Oct	JQ820473		
7	El Gezira	Oct	JQ820480	JQ820487	
28	El Gezira	Oct	JQ820474	JQ820491	JQ820486
30	El Gezira	Oct	JQ820481		
34	El Gezira	Oct	JQ820475		
85	El Gezira	Oct	JQ820476	JQ820488	JQ820485
86	El Gezira	Oct	JQ820477	JQ820489	JQ820484

*Samples collected at some point during October–December but exact month of collection not known. †Blank cells indicate no sequences.

### Phylogenetic Analysis

**Figure 3:**
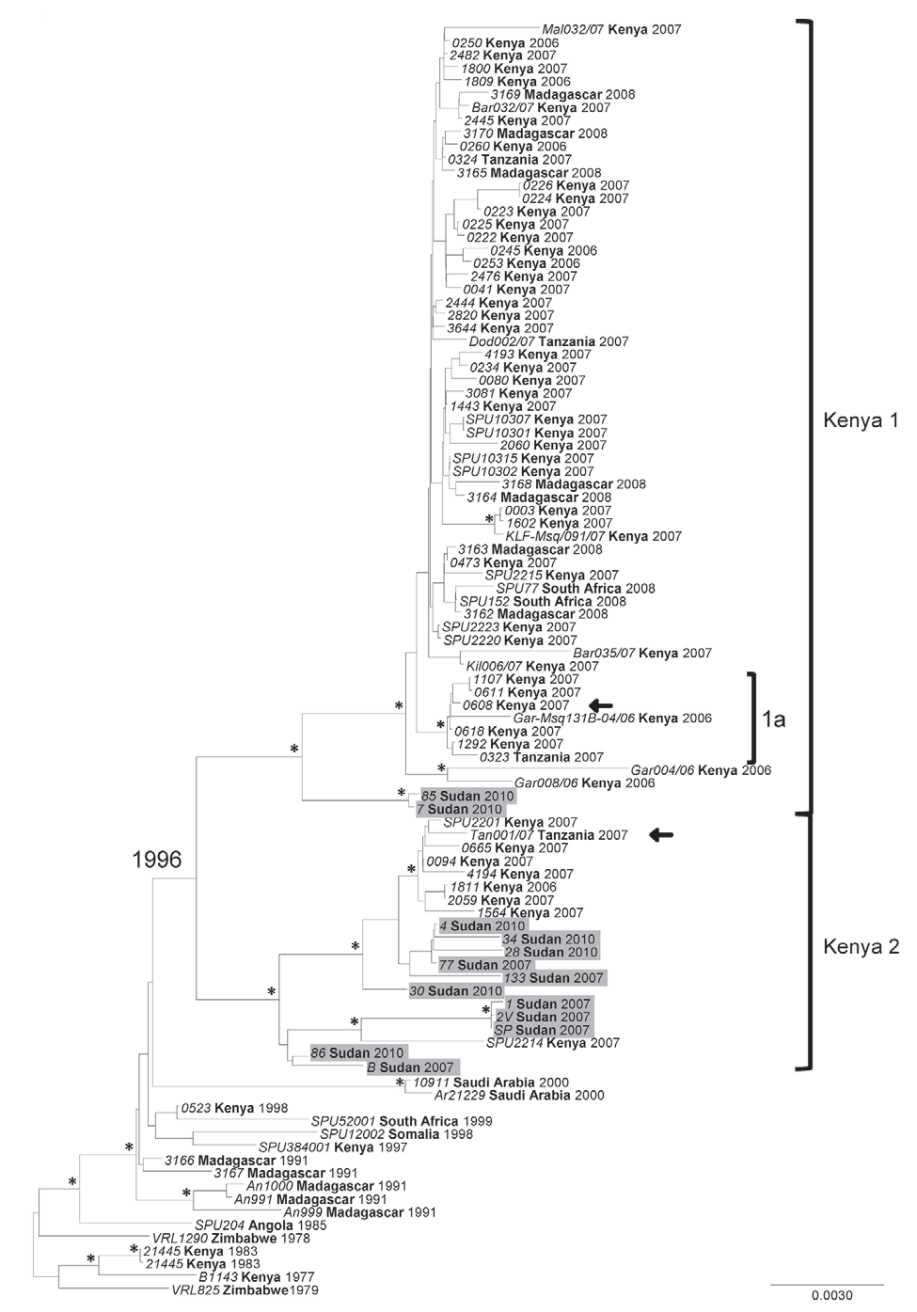
Phylogenetic analysis of complete Rift Valley fever virus S (small) segment sequences represented as an abbreviated maximum clade credibility tree. Asterisk indicates nodes with highest posterior density >0.95. Sudan sequences are shaded. Arrow indicates reassortant viruses. Scale bar represents substitutions per site per year. The complete tree is presented in Technical Appendix Figure 1. Country names appear in **boldface**, and strain names appear in *italics*.

**Figure 4:**
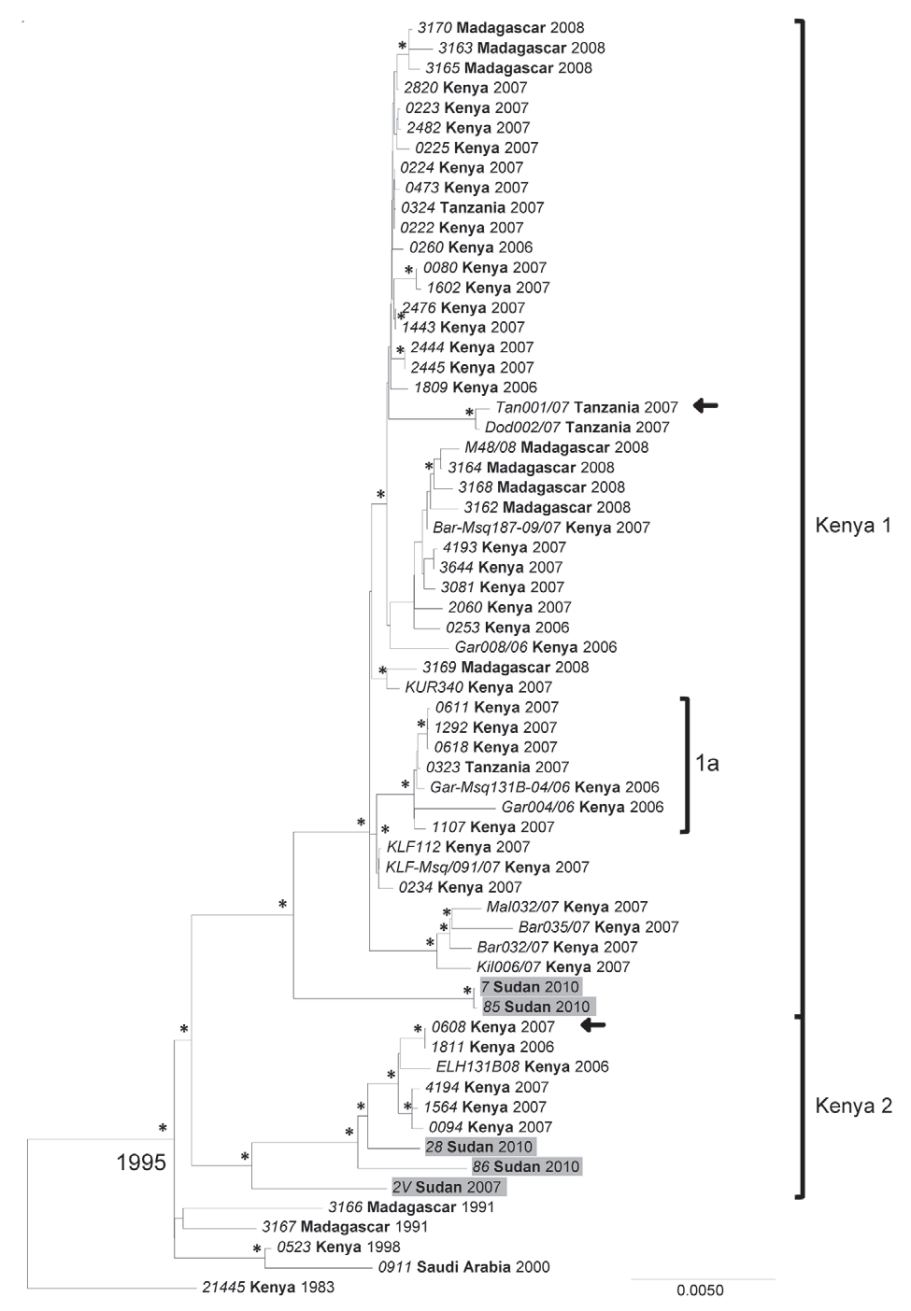
Phylogenetic analysis of complete Rift Valley fever virus M (medium) segment sequences represented as an abbreviated maximum clade credibility tree. Asterisk indicates nodes with highest posterior density >0.95. Sudan sequences are shaded. Arrow indicates reassortant viruses. Scale bar represents substitutions per site per year. The complete tree is presented in Technical Appendix Figure 2. Country names appear in **boldface**, and strain names appear in *italics*.

**Figure 5:**
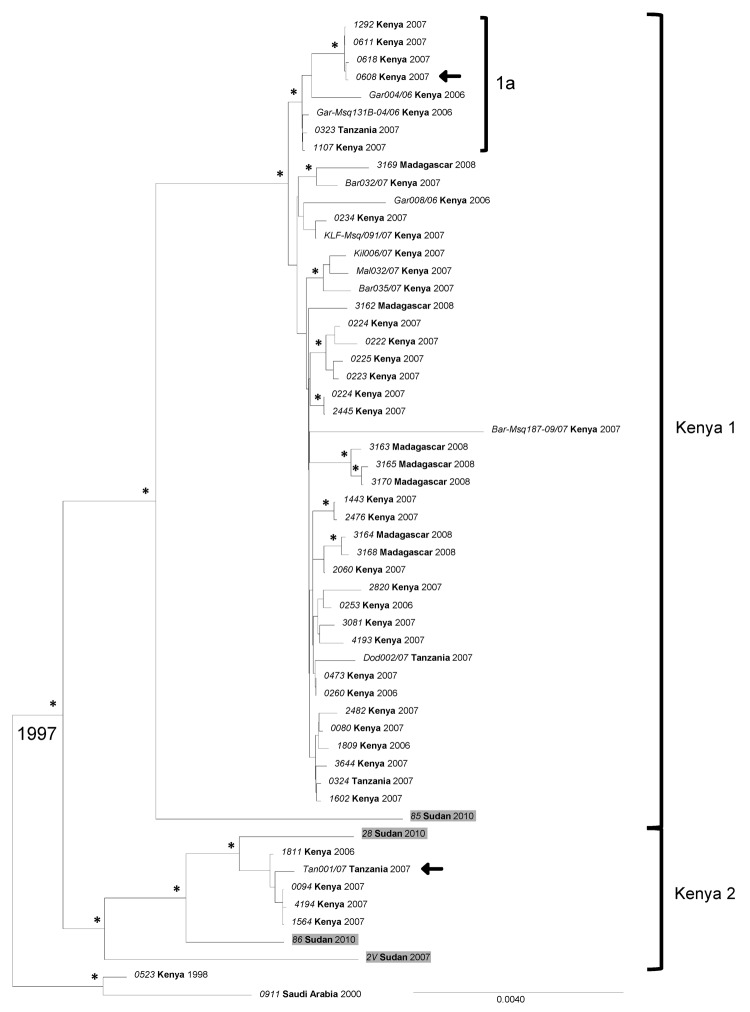
Phylogenetic analysis of complete Rift Valley fever virus L (large) segment sequences represented as an abbreviated maximum clade credibility tree. Asterisk indicates nodes with highest posterior density >0.95. Sudan sequences are shaded. Arrow indicates reassortant viruses. Scale bar represents substitutions per site per year. The complete tree is presented in Technical Appendix Figure 3. Country names appear in **boldface**, and strain names appear in *italics*.

When conducting the phylogenetic analysis to compare the complete RVFV segment sequences available from GenBank to sequences generated in this study, the S, M, and L RNA segment datasets were analyzed separately. Previously, 2006–2007 outbreak samples from Kenya, Tanzania, and Madagascar were grouped into a genetic lineage comprising 2 sublineages referred to as Kenya-1 and Kenya-2. The analyses placed all 3 segments of the Sudan strains into either of the 2 Kenya sublineages with no evidence of reassortment (Figures 3,4,5; Technical Appendix). We found no link relative to the genetically more distant Egypt RVFV strains detected during the 1970s and 1994. All Sudan RVFV strains from the 2007 outbreak, and several strains from the 2010 RVF outbreak, were embedded into the Kenya-2 sublineage; however, 2 strains from 2010 were included in the Kenya-1 sublineage. Of the 7 states where suspected RVF cases were distributed, whole-genome sequences were generated from case-patients from Khartoum, White Nile, and El Gezira. The Khartoum strains grouped with strains from El Gezira and White Nile States (Figures 3,4,5).

When we included additional Tanzania RVFV sequences from GenBank in the analyses, 1 strain, TAN/tan-001/07, fell in the Kenya-2 sublineage for the S and L segments and in the Kenya-1 sublineage for the M segment. These findings added another reassortant to the example (Kenya strain, #0608) (Figures 3,4,5) (*26*,*37*).

The mean molecular evolutionary rates with 95% highest posterior density intervals were calculated for each segment and were similar to those previously reported (*25*,*26*): 4.20 × 10^−4^ (3.09 × 10^−4^ to 5.38 × 10^−4^) nt substitutions per site per year for the S segment; 5.06 × 10^−4^ (3.27 × 10^−4^ to 6.76 × 10^−4^) nt substitutions per site per year for the M segment; and 4.29 × 10^−4^ (2.65 × 10^−4^ to 6.03 × 10^−4^) nt substitutions per site per year for the L segment. The calculations for the time to most recent common ancestor (MRCA) for all included strains were also similar to previous analyses (*25*,*26*): 91.98, 114.17, and 108.95 years for the S, M, and L segments, respectively. When the 2007 and 2010 outbreak lineages were examined more closely, the MRCA dated to 1996, 1995, and 1997 for the S, M, and L segments, respectively.

## Discussion 

In October 2007, RVF was detected in the several states of Sudan that border the White Nile River (*20*). Virus activity was detected in 7 states: Nile, Khartoum, Kassala, El Gezira, White Nile, Sennar, and Upper Nile; large numbers of human infections occurred during the relatively short period of 2 months (Figure 1). The substantial RVF outbreak was first detected in Kenya and Tanzania in late 2006 to early 2007 after a season of heavy rainfall (*21*). RVFV activity continued for several years and covered a large geographic area, including Sudan in 2007, and South Africa and Madagascar in 2008 (*1*,*20*,*25*,*38*,*39*).

Although substantial rainfall events were most likely the major cause of spread and maintenance of the RVF outbreak (*21*,*40*), the contribution of irrigation projects is less well understood. Several recent studies have examined the effects of irrigation and agricultural practices on mosquito populations (*41*–*44* in online Technical Appendix). However, the effect of these agricultural processes on RVF outbreaks and maintenance during interepidemic periods has not been directly studied. The large RVF outbreak that occurred in the regions surrounding the Senegal River during 1987–1988 was thought to be linked to completion of the Maka-Diama dam built in 1986 and the extensive irrigation system developed at this time (*45* in online Technical Appendix). The potential effect of irrigation on RVF should be considered, since new industries in the Sudan are changing the landscape in several RVF-affected states. El Gezira and, to a lesser degree, Sennar, White Nile, and Khartoum States, have vast tracts of irrigated land. Khartoum State has a growing agricultural industry along the Blue Nile River, particularly in Hilat Kuku, Khartoum North, which was the focus of the 1977 Sudan RVF outbreak (*15*). During the end of November 2007, the El Gezira authorities instituted an extensive insecticide spraying program and the federal government restricted trade of livestock and associated products in the state, which may have contributed to the subsequent decline in suspected cases. Future studies would be useful for determining appropriate vector control strategies in irrigated areas.

The epidemiologic data for the 156 suspected RVF cases of 2007 indicated that more male than female case-patients fit the case definition for RVF; however, the percentage of confirmed cases was equivalent for both sexes. The mean age of suspected case-patients was 28 years, consistent with reports from Kenya and Tanzania, where persons 20–30 years old were most affected (*27*,*29*). Young adult men in these affected regions are generally more exposed than women to potentially infected mosquitoes during agricultural work or direct contact with viremic livestock and potentially infected livestock by-products, such as aborted fetal material and raw milk products.

Suspected case-patients were from the 7 states listed previously; most were from Khartoum, Kassala, El Gezira, and White Nile (Figure 1). Rainfall or the new irrigation schemes mentioned previously might have influenced case distribution. Although the overall percentage of confirmed cases was 50%, the percentage of confirmed cases in each state ranged from 15% to 84% (data not shown). The variation also could result from different interpretations of the case definition or increases in other febrile illnesses affected by weather conditions similar to those affecting RVF (e.g., an increase in disease vectors). The possibility of reporting bias also exists because patients can be referred to medical centers in neighboring states.

In 2010, RVF cases were again detected in El Gezira State. The outbreak was first characterized by abortions in ewes and does and followed by infections in persons with histories of contact with aborted fetal material (I.E. Aradaib, pers. comm.). Unfortunately, detailed information about the 100 suspected case-patients tested was not available for analysis.

RVFV-positive samples from several states of Sudan were selected for full-genome analysis to determine the relationship of the strains circulating in Sudan to other known strains identified globally, especially those from the 2006–2007 outbreaks in Kenya and Tanzania and from Egypt during the 1970s and in 1994. A total of 13 complete S segment, 5 complete M segment, and 4 complete L segment sequences were obtained. Phylogenetic analysis of these sequences identified several RVFV variants circulating during the Sudan outbreaks and placed them all in the large lineage containing the Kenya-1 and Kenya-2 sublineages, which defined the eastern Africa outbreak in 2006–2008 (*26*). No genetic relationship was found relative to the earlier Egypt strains. Previously, only RVFV strains identified in Kenya were embedded in the Kenya-2 sublineage; however, as more sequences become available, this sublineage clearly has also become widely geographically distributed (*25*,*26*). Bayesian analysis was used to help elucidate whether RVFV diversity in Sudan resulted from multiple introductions or from acquired changes over time from a single introduction event. Several observations indicate that multiple introductions of RVFV occurred as part of its spread from eastern Africa since the 1996–1997 RVF outbreak (*46* in Technical Appendix). The date to the MRCA for the overall Kenya lineage is circa 1996, and the MRCA for the 2007 and 2010 Sudan sequences also dates to 1996 instead of 2007. The closest MRCA between the 2007 and 2010 sequences is 5 years (2005). Dating of the MRCA of the overall Kenya lineage concurs with the MRCA of previous studies, which supports the robustness of the models chosen for analysis, even considering the limitations of sample size, collection methods, and environmental factors (*25*,*26*). Identical or nearly identical sequences were identified for different states and years, Khartoum in 2007 and El Gezira in 2010, as well as Khartoum and West Nile in 2007. These sequences indicate recent movement of the virus in this region and support the necessity and utility of surveillance systems for recognizing when and where a large epidemic is imminent. Understanding where the virus is circulating during interepidemic periods can make it easier to interpret data from prediction tools (*21*) and focus preventive measures, such as vector control, livestock vaccination, and education campaigns, on high-risk areas.

The significance of detecting an additional M segment reassortant remains unclear. Reassortment seems to be a relatively rare event because only 2 reassortants were detected for the 54 complete genome sequences (S, M, and L) from the 2006–2007 RVF outbreak. However, it does support cocirculation of both Kenya sublineages in the same geographic location.

The addition of RVFV sequences from Sudan enhances our understanding of the expansion and, to some degree, maintenance of the virus during a large epidemic and the interepidemic period that follows. The ability to sequence entire viral genomes relatively quickly should lead to rapid progress in understanding the detailed ecology of RVFV. Ongoing surveillance and RVFV characterization also should help determine the pattern of virus maintenance between epizootic events. As prediction tools become more accurate and available, these data will provide public health authorities an opportunity to anticipate and prepare for RVF outbreaks.

Technical Appendix Supplemental references and phylogenetic analyses of complete Rift Valley fever virus small, medium, and large segment sequences as represented as maximum clade credibility trees.
