# Supplementary material for: Rift Valley Fever, Sudan, 2007 and 2010
Source: Emerg Infect Dis. 2013 Feb;19(2):246–53. doi: 10.3201/eid1902.120834 (PMC3559046; doi:10.3201/eid1902.120834)
Supplement: Technical Appendix — Supplemental references and phylogenetic analyses of complete Rift Valley fever virus small, medium, and large segment sequences as represented as maximum clade credibility trees. [file 12-0834-Techapp-s1.pdf]

Article DOI: http://dx.doi.org/10.3201/eid1902.120834

Rift Valley Fever, Sudan, 2007 and 2010

Technical Appendix

Supplemental References

41. Dia I, Ba H, Mohamed SAO, Diallo D, Lo B, Diallo M. Distribution, host preference and infection rates of malaria vectors in Mauritania. Parasit Vectors. 2009;2:61. PubMed http://dx.doi.org/10.1186/1756-3305-2-61

42. Muturi EJ, Shililu J, Jacob B, Gu W, Githure J, Novak R. Mosquito species diversity and abundance in relation to land use in a Riceland agroecosystem in Mwea, Kenya. J Vector Ecol. 2006;31:129–37. PubMed http://dx.doi.org/10.3376/1081-1710(2006)31[129:MSDAAI]2.0.CO;2

43. Muturi EJ, Shililu JI, Gu W, Jacob BG, Githure JI, Novak RJ. Larval habitat dynamics and diversity of *Culex* mosquitoes in rice agro-ecosystem in Mwea, Kenya. Am J Trop Med Hyg. 2007;76:95–102. PubMed

44. Schäfer ML, Lundström JO. Different responses of two floodwater mosquito species, *Aedes vexans* and *Ochlerotatus sticticus* (Diptera: Culicidae), to larval habitat drying. J Vector Ecol. 2006;31:123–8. PubMed http://dx.doi.org/10.3376/1081-1710(2006)31[123:DROTFM]2.0.CO;2

45. Thonnon J, Picquet M, Thiongane Y, Lo M, Sylla R, Vercruysse J. Rift Valley fever surveillance in the lower Senegal River basin: update 10 years after the epidemic. Trop Med Int Health. 1999;4:580–5. PubMed http://dx.doi.org/10.1046/j.1365-3156.1999.00437.x

46. Woods CW, Karpati AM, Grein T, McCarthy N, Gaturuku P, Muchiri E, et al. An outbreak of Rift Valley fever in northeastern Kenya, 1997–98. Emerg Infect Dis. 2002;8:138–44. PubMed http://dx.doi.org/10.3201/eid0802.010023

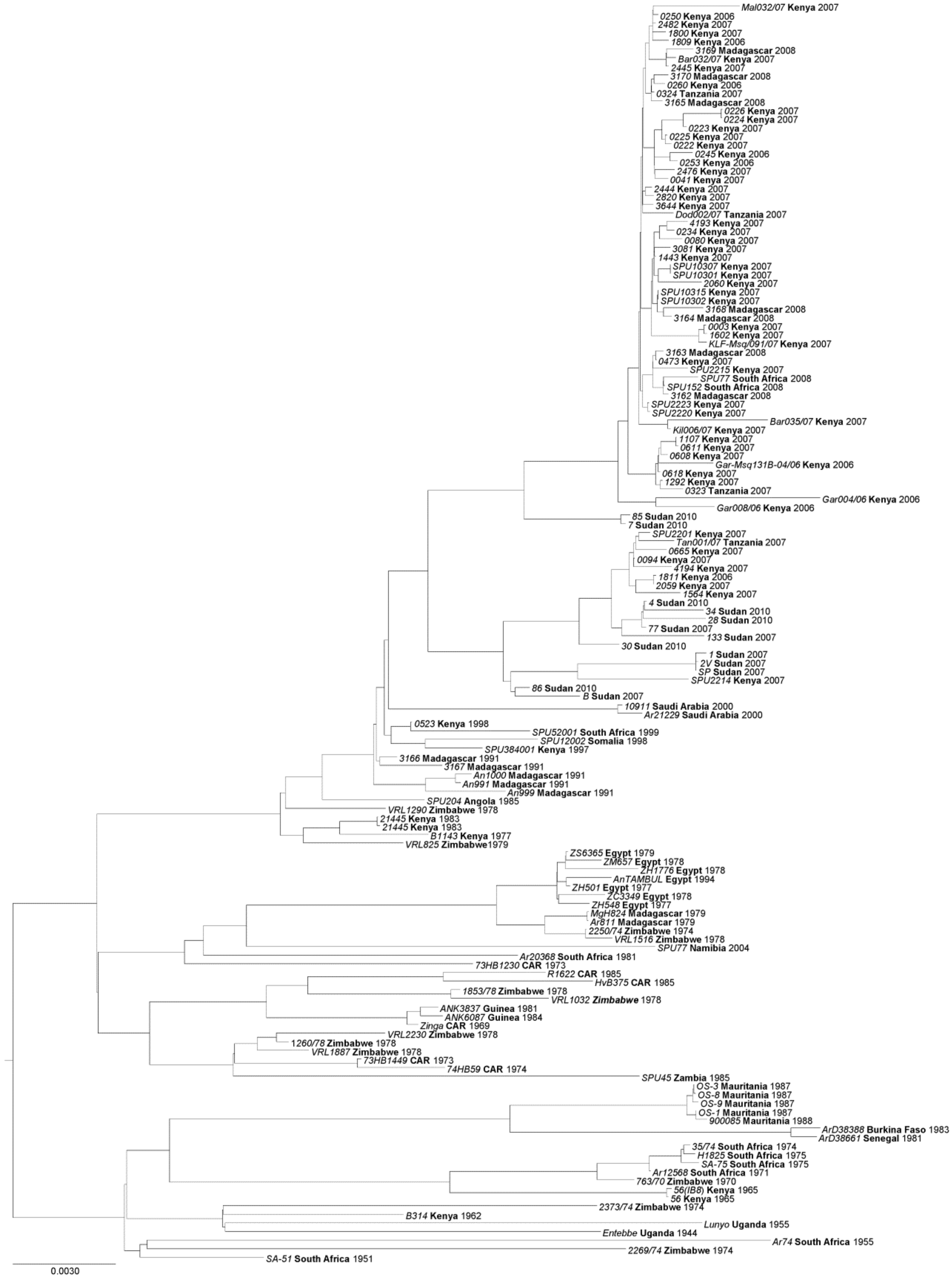

Technical Appendix Figure 1. Phylogenetic analysis of complete Rift Valley fever virus S (small) segment sequences represented as a maximum clade credibility tree. Scale bar represents substitutions per site per year.

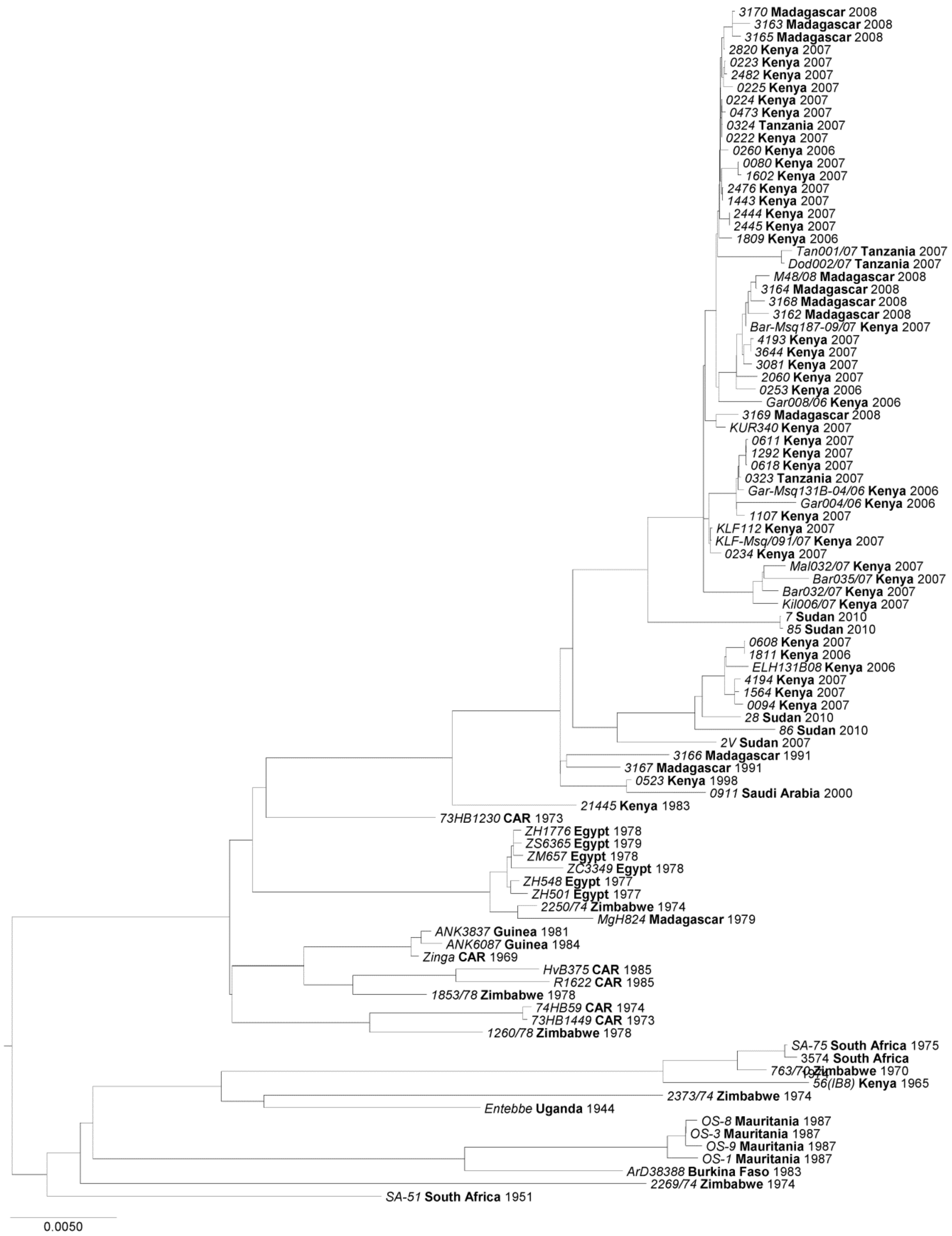

Technical Appendix Figure 2. Phylogenetic analysis of complete Rift Valley fever virus M (medium) segment sequences represented as a maximum clade credibility tree. Scale bar represents substitutions per site per year.

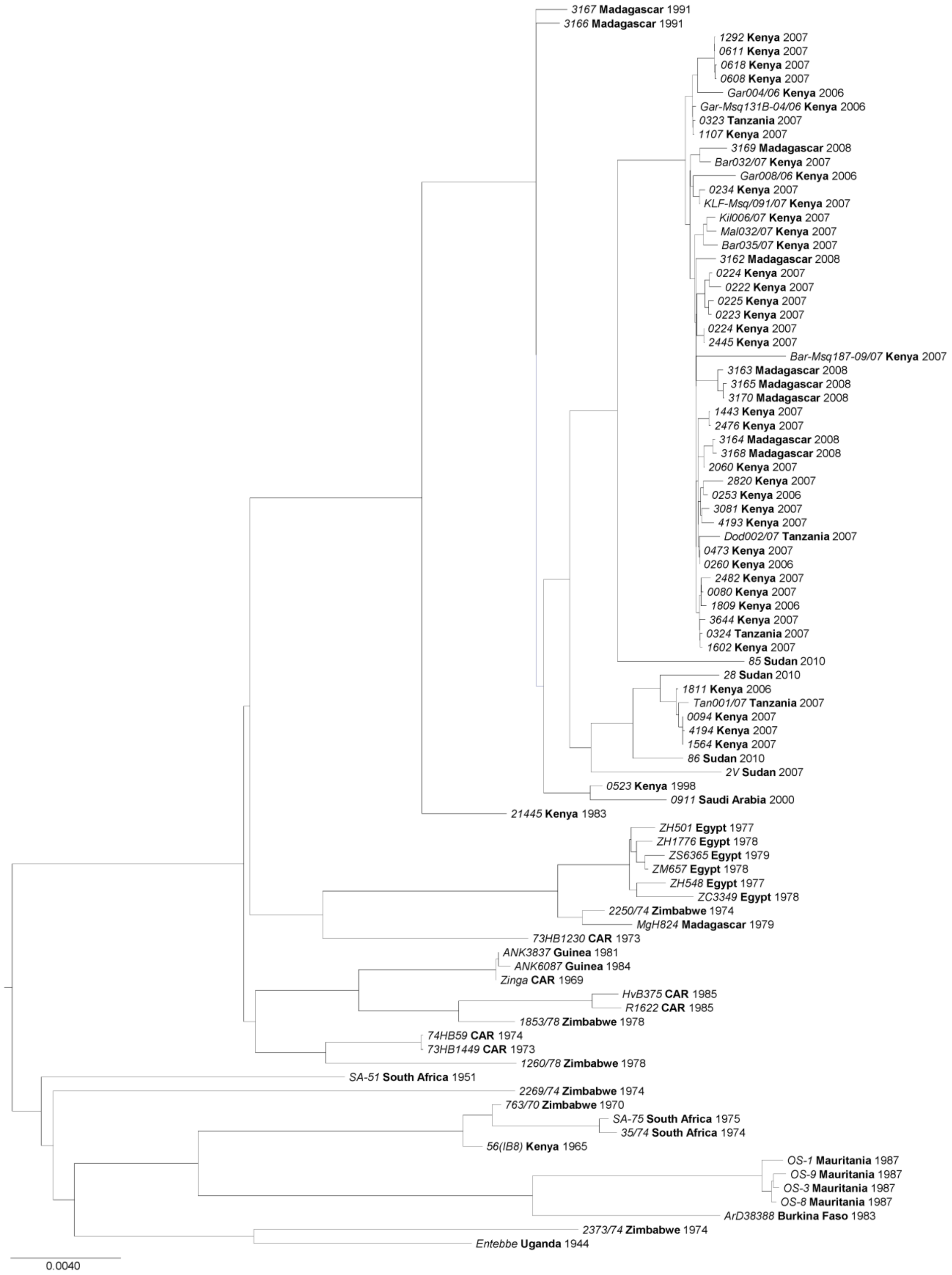

Technical Appendix Figure 3. Phylogenetic analysis of complete Rift Valley fever virus L (large) segment sequences represented as a maximum clade credibility tree. Scale bar represents substitutions per site per year.
